# Not all anthocyanins are born equal: distinct patterns induced by stress in *Arabidopsis*

**DOI:** 10.1007/s00425-014-2079-1

**Published:** 2014-06-06

**Authors:** Nik Kovinich, Gilbert Kayanja, Alexandra Chanoca, Ken Riedl, Marisa S. Otegui, Erich Grotewold

**Affiliations:** 1 Center for Applied Plant Sciences (CAPS), The Ohio State University, 012 Rightmire Hall, 1060 Carmack Road, Columbus, OH 43210 USA; 2 Departments of Molecular Genetics and Horticulture and Crop Sciences, The Ohio State University, 012 Rightmire Hall, 1060 Carmack Road, Columbus, OH 43210 USA; 3 Department of Food Science and Technology, The Ohio State University, 110 Parker Food Science and Technology Building, 2015 Fyffe Road, Columbus, OH 43210 USA; 4 Department of Botany, University of Wisconsin, B119 Birge Hall, 430 Lincoln Drive, Madison, WI 53706 USA; 5 Department of Genetics, University of Wisconsin-Madison, Madison, WI 53705 USA

**Keywords:** Abiotic stress, Anthocyanin pigmentation, Flavonoid

## Abstract

**Different abiotic stress conditions induce distinct sets of anthocyanins, indicating that anthocyanins have different biological functions, or that decoration patterns of each anthocyanin are used for unique purposes during stress.**

The induction of anthocyanin accumulation in vegetative tissues is often considered to be a response of plants to biotic or abiotic stress conditions. *Arabidopsis thaliana* (Arabidopsis) accumulates over 20 anthocyanins derived from the anthocyanidin cyanidin in an organ-specific manner during development, but the anthocyanin chemical diversity for their alleged stress protective functions remains unclear. We show here that, when grown in various abiotic stress conditions, *Arabidopsis* not only often accumulates significantly higher levels of total anthocyanins, but different stress conditions also favor the accumulation of different sets of anthocyanins. For example, the anthocyanin patterns of seedlings grown at pH 3.3 or in media lacking phosphate are very similar and characterized by relatively high levels of the anthocyanins A8 and A11. In contrast, anthocyanin inductive conditions (AIC) provided by high sucrose media are characterized by high accumulation of A9* and A5 relative to other stress conditions. The modifications present in each condition correlate reasonably well with the induction of the respective anthocyanin modification enzymes. Taken together, our results suggest that *Arabidopsis* anthocyanin profiles provide ‘fingerprints’ that reflect the stress status of the plants.

**Electronic supplementary material:**

The online version of this article (doi:10.1007/s00425-014-2079-1) contains supplementary material, which is available to authorized users.

## Introduction

Anthocyanins are flavonoid pigments responsible for many of the red, violet and purple colors characteristic of fruits and flowers, where they function as attractants for pollinators or seed-dispersing organisms (Grotewold 2006). In many plant species, anthocyanins accumulate transiently in the epidermal cell layer of vegetative tissues at specific stages of development, such as leaf expansion (Parkin 1903), likely playing a role in photoprotection (Hatier and Gould 2009). However, abiotic stresses can induce anthocyanin synthesis in the chlorenchyma cells of the leaves of most plant species (Parkin 1903). The function of stress-induced anthocyanins is presently not known; one prominent hypothesis is that they serve as antioxidants that quench ROS (reviewed by Gould 2004a; Hatier and Gould 2009; Agati et al. 2012). ROS are mainly produced in chloroplasts and mitochondria via the aerobic reactions of photosynthesis and respiration, and accumulate to relatively high levels under stress conditions that limit photosynthesis (Mittler 2002; Rhoads et al. 2006). Anthocyanins are mainly sequestered in vacuoles, however, the enzymes of flavonoid biosynthesis are believed to be localized mainly on the cytosolic face of the ER, anchored to the membrane by cytochrome P450s such as flavonoid 3′-hydroxylase (F3′H) (Winkel 2004). Despite the different subcellular localizations of anthocyanins and ROS, anthocyanin-containing leaf cells have been shown to exhibit greater capacity to remove H_2_O_2_ than cells that lack these compounds (Gould et al. 2002).

Abiotic stresses that induce anthocyanin synthesis include drought in rice and *Arabidopsis* (Basu et al. 2010; Sperdouli and Moustakas 2012), cold in maize, *Arabidopsis*, and citrus (Christie et al. 1994; Crifò et al. 2011), high salt in tomato and red cabbage (Eryılmaz 2006), nutrient deficiency in *Arabidopsis*, hibiscus, and carrot (Mizukami et al. 1991; Rajendran et al. 1992; Jiang et al. 2007), osmotic stress in carrot callus and grapevine cell cultures (Rajendran et al. 1992; Suzuki 1995), and exposure to low pH of the medium in strawberry suspension cell cultures (Zhang and Furusaki 1997; reviewed by Chalker-Scott 1999; Winkel-Shirley 2002). The presence of sucrose in the culture medium also induces anthocyanin synthesis by a mechanism dependent on the MYB transcription factor, PAP1 (Teng et al. 2005; Solfanelli et al. 2006). PAP1 was demonstrated to be a major regulator of anthocyanin synthesis, as its overexpression by cauliflower mosaic virus 35S enhancer resulted in induction of anthocyanin genes and massive ectopic accumulation of anthocyanins (Borevitz et al. 2000; Tohge et al. 2005). As a result of PAP1 induction by sucrose, an artificial culturing condition consisting of 3 % sucrose and high light, termed anthocyanin induction condition or AIC, has been extensively used for the research of anthocyanin biosynthesis and trafficking (Poustka et al. 2007; Pourcel et al. 2010).

Recently, direct evidence has emerged that under drought and other oxidative stresses, plants engineered to produce high levels of anthocyanins have increased yield and antioxidant capacity compared to control plants (Nakabayashi et al. 2013; Wang et al. 2013). These observations are expected to spur the engineering of anthocyanins in crop plants for increased antioxidant capacity. In addition, unrelated efforts to engineer color into commercial, genetically modified commodities to facilitate their identification and monitoring (Kovinich et al. 2011), and anthocyanin content in foods for added health benefits (Butelli et al. 2008), underscore the importance of determining whether all anthocyanins may be considered equal in terms of their function in the plant.

**Fig. 1:**
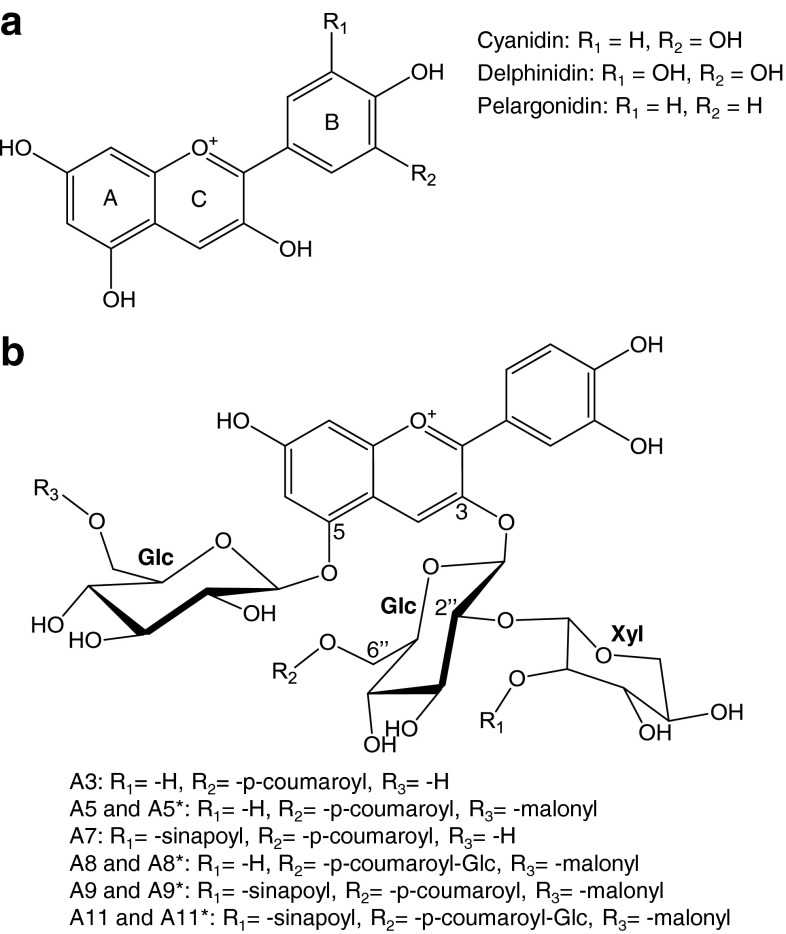
Anthocyanin structure. Common anthocyanidin backbones (**a**). *Arabidopsis* anthocyanins analyzed in this study (**b**). *Asterisk* indicates a tautomer. See (Pourcel et al. 2010; Saito et al. 2013; Tohge et al. 2005) for the complete list of *Arabidopsis* anthocyanins

Collectively, plants produce more than 500 anthocyanins with unique chemical structure (Andersen and Markham 2006), and individual anthocyanins possess different radical scavenging activity in vitro (Garcia-Alonso et al. 2005). Anthocyanins are characterized by the degree of hydroxylation or methoxylation of the anthocyanidin chromophore, and the decorations added to this backbone. For example, pelargonidin, cyanidin, and delphinidin contain one-, two- and three hydroxyl groups on the B-ring, respectively (Fig. 1a). The anthocyanidin core becomes a stable anthocyanin by the addition of a glycose (mainly glucose) at C3; however, acyl, hydroxycinnamic acid, and other moieties can be added to the backbone to yield more complex anthocyanins. It is common for plants to accumulate several different types of anthocyanins that derive from one or more anthocyanidin precursors. *Arabidopsis* accumulates more than 20 highly decorated derivatives of cyanidin (Tohge et al. 2005; Pourcel et al. 2010; Saito et al. 2013); the structures of those discussed in this study are illustrated in Fig. 1b.

The genes required for the biosynthesis and regulation of anthocyanins and other flavonoids are well described (Koes et al. 1994, 2005; Mol et al. 1998; Winkel-Shirley 2001; Grotewold 2006; Petroni and Tonelli 2011a; Saito et al. 2013). The inducible accumulation of anthocyanins in the mesophyll is controlled by the homeodomain transcription factor *ANTHOCYANINLESS2* (Kubo et al. 1999); however, it remains to be determined whether *ANTHOCYANINLESS2* activates anthocyanin biosynthetic genes directly, or causes the induction of the MYB-bHLH-WD40 ternary complex, which includes the MYB transcription factor PAP1, known to bind and activate the promoters of anthocyanin genes (Petroni and Tonelli 2011b). In *Arabidopsis*, the sequential order of biosynthetic steps of anthocyanin synthesis is best represented by a grid, whereby a single product of one reaction may be used as a substrate for several different reactions (Yonekura-Sakakibara et al. 2012). The enzymes that determine the pathway that intermediates traverse through the grid as well as the identity of the final products are; 5GT (a.k.a UGT75C1), BLGU10, A5GlcMalT, A3G2″XylT (a.k.a. UGT79B1), A3GlcCouT, and SAT (a.k.a. A3Glc2″XylSinT) (Tohge et al. 2005; D’Auria et al. 2007; Fraser et al. 2007; Yonekura-Sakakibara et al. 2012; Miyahara et al. 2013). Specific anthocyanins accumulate during development in an organ-specific manner in *Arabidopsis* (Saito et al. 2013). The synthesis of numerous structurally diverse anthocyanins and their presence in different organs may suggest that all anthocyanins may not be considered equal, and in light of this an important question remains––*do different anthocyanins accumulate in response to different stress conditions*?

## Materials and methods

### Plant materials and growth conditions

Wild-type seeds of *Arabidopsis thaliana* (ecotype Columbia) were surface-sterilized on a mixer wheel for five min in 70 % ethanol 0.2 % Triton X, rinsed 3 times with ethanol, dried, and planted on 0.5MS/3 % sucrose/0.5 % agar medium. After 3 days of stratification, plants were grown for 10 days under 24 h white light at 22 °C for control condition. For stress conditions, specific additives were combined with the medium, and pH adjusted to 5.8 before autoclaving. For experiments done in AIC seeds were sown in water containing 3 % sucrose and grown on a rotary shaker under the same conditions as above for 5 days.

### Stress conditions and metabolite extraction

The following concentrations were applied for stress conditions: 250 mM mannitol, 100 mM NaCl, 100 mM MgSO_4_, low pH was adjusted to 3.3 (not completely a gel as a consequence of the low pH) and high pH to 7.3. For cold treatment, plants were transferred 8 days after germination to 4 °C, under the same light conditions, for 48 h before tissue collection. For the condition −P, 0.5MS medium was prepared using −P MS (Caisson Labs MSP11). The control was plants grown on 0.5MS under the same light and temperature conditions as the stress treatments.

Ensuring that roots did not have any residual medium, whole seedlings were collected and stored at −80 °C until lyophilization. After 2 days lyophilization, dry weight was measured and 50 µg/μl extraction solution [50 % (v/v) methanol, 3 % (v/v) formic acid] was added and incubated at room temperature overnight on a rotary shaker. Tubes were then centrifuged at 13,500*g* for 2 min and the supernatant was passed through 0.2 µM filters (Nanosep ODM02C35), and the filtrate analyzed by spectrophotometry and HPLC–PDA.

### Metabolite analysis

Total anthocyanins were measured using a spectrophotometer (Nano Drop ND-1000). Metabolite compositions were analyzed using a Waters Alliance 2695 HPLC equipped with PDA. 20 μl of plant extract was injected onto a Symmetry C18 column (4.6 × 75 mm, 100Å, 3.5 µm) held at 35 °C. The mobile phase flow rate was 1 mL min^−1^ and consisted of buffers A [5 % (v/v) formic acid in water] and B [5 % (v/v) formic acid in acetonitrile], with the following elution profile (0 min 100 % A, 20 min 75 % A, 22 min 20 % A, 22.1 min 100 % B, 25 min 100 % B, 25.1 min 100 % A, 32 min 100 % A) using a linear gradient between time points. Area under the peak (AU^2^) was determined using the manual integration option of Empower software, at 532 nm for anthocyanins, and 330 nm for SEs and flavonols. Metabolite identities were determined by LC–MS/MS as described previously (Pourcel et al. 2010). To determine the extinction coefficients of A11 and A9* relative to cyanidin, *Arabidopsis* anthocyanins were first purified by HPLC–PAD equipped with a Waters Fraction Collector II. The purity of isolates was validated by TLC and by HPLC–PAD monitoring at 532, 330, and 280 nm. To determine extinction coefficients, absorbances of individual compounds, exposed or not to acid hydrolysis, were compared at 530 nm, and extinction coefficient of the hydrolyzed sample was assigned the value of cyanidin in solvent 0.1 % HCl in ethanol (34700 L cm^−1^ mol) (Ribereau-Gayon 1959). Acid hydrolysis was conducted using seven volumes of 2:3 HCl:1-butanol for 15 min at 95 °C, compounds were lyophilized to dryness and resuspended in 0.1 % HCl in ethanol. To confirm complete hydrolysis, TLC was conducted according to Andersen and Francis (1985) using cellulose layer and the solvent system 24.9:23.7:51.4 (HCl:formic acid:water, by vol.). The commercial standards cyanidin and cyanidin 3-*O*-glucoside were used as controls.

### Cluster analysis

Cluster analysis was performed with Multiexperiment Viewer software Version 4.9 using default parameters and the Euclidean Distance metric. Metabolite profiles were obtained as described above. Gene expression data were obtained from the Bio-Analytic Resource (http://www.bar.utoronto.ca/efp).

## Results and discussion

### Anthocyanin induction by different abiotic stress conditions

**Fig. 2:**
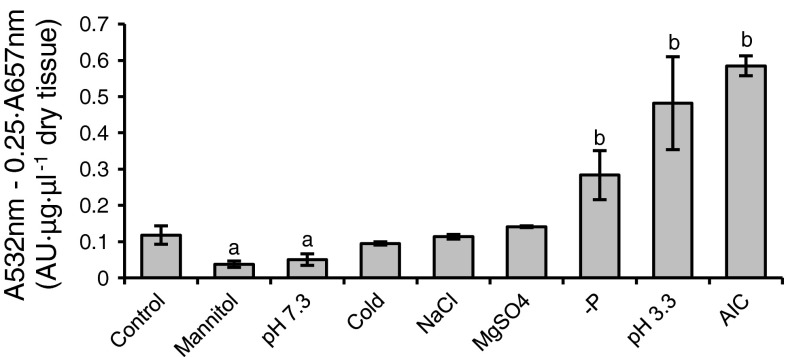
Amount of total anthocyanins produced by *Arabidopsis* grown in various stress conditions. Plants were cultured under stress conditions, tissues were extracted, and metabolites analyzed as described in the “Materials and methods”. *Error bars* represent the standard error of the mean (*n* = 3). ^a^Less than control, ^b^greater than control, *P* < 0.05; two-tailed Student’s *t* test

Anthocyanins are commonly reported as being induced by abiotic stress. However, the level of induction of anthocyanins across different stresses is unknown. To determine the response of *Arabidopsis* from the perspective of anthocyanin accumulation, we grew *Arabidopsis* under seven physiologically extreme stress conditions previously reported to trigger anthocyanin accumulation, and the levels of total anthocyanin were quantified by spectrophotometry at 532 nm (Fig. 2). For reference, we also included seedlings grown for 5 days in AIC, an artificial liquid culture condition that does not represent a natural physiological stress, but is well characterized for inducing high levels of anthocyanins (Poustka et al. 2007; Pourcel et al. 2010). Our results show that seedlings grown on the 0.5MS control condition for 10 days exhibited some low-level anthocyanin pigmentation, similar to that reported previously for 3-day-old *Arabidopsis* seedlings (Shirley et al. 1995). Relative to the control, deficiency of the macronutrient phosphate (−P) and low pH medium (pH 3.3) resulted in significant induction of total anthocyanin levels, similar to AIC (Fig. 2). It is noteworthy that AIC media contains 3 % sucrose, similar to the control media, but lacks other nutrients such as a nitrogen source, which has been shown to further enhance the accumulation of anthocyanins (Hsieh et al. 1998). Under our experimental conditions, 100 mM NaCl or 100 mM MgSO_4_ did not result in a statistically significant change in the levels of total anthocyanin. This contrasts the induction of anthocyanins observed in 7-day-old tomato and red cabbage seedlings after the application of 100 mM NaCl through a hydroponic system (Eryılmaz 2006), and may simply be due to reduced uptake of salts from our agar-based media, or to adaptation to the stress over longer-term exposure, rather than different responses among species. Unexpectedly, 250 mM mannitol resulted in a statistically significant (*P* < 0.05, two sided *t* test) reduction in total anthocyanins, as did pH 7.3. High pH has also been shown to reduce total anthocyanin levels in grape cell cultures (Suzuki 1995). By contrast, seedlings grown in pH 7.3 medium had unchanged levels of flavonols and SEs, as indicated by the absence of a change in the absorbance at 330 nm, whereas growth in mannitol led to a reduction in both flavonol and SE absorbance (Supplemental Fig. S1). Overall, our results demonstrate that similar to AIC, low pH and phosphate deficiency induce anthocyanin accumulation, whereas osmotic stress with mannitol and high pH promoted a reduction in total anthocyanins.

### Unique anthocyanin profiles result from different stress conditions

*Arabidopsis* synthesizes more than 21 anthocyanins when cultured in AIC or when overexpressing the transcription factor *PAP1* (Tohge et al. 2005; Rowan et al. 2009; Pourcel et al. 2010; Saito et al. 2013). Our results show that, under our experimental growth conditions, some stress conditions previously shown to induce anthocyanin pigmentation (e.g., salt, cold) did not result in significantly elevated total levels of anthocyanins (Fig. 2). Previous studies suggested that *Arabidopsis* anthocyanins may have different rates of catabolism (Rowan et al. 2009), hence, similar levels of anthocyanins could be a consequence of some being induced by a particular stress condition, while others are catabolized, or their synthesis repressed. This raises the question of whether specific anthocyanins may be preferentially induced, or repressed, in response to a particular stress.

**Fig. 3:**
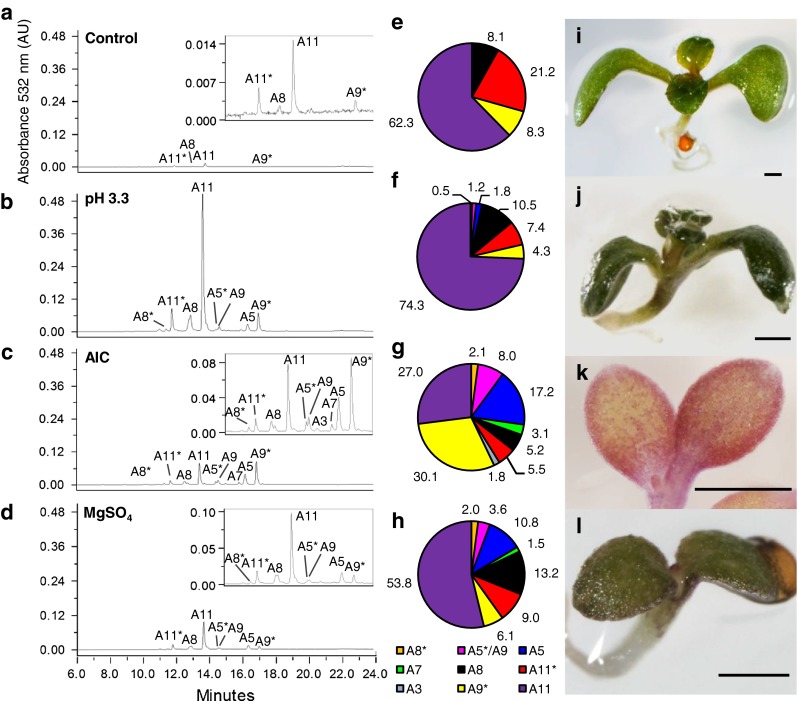
Anthocyanin compositions from *Arabidopsis* grown in stress conditions. HPLC–PDA chromatograms of aqua-methanol extracts (**a**–**d** *insets* are chromatograms at full *scale*), percentage of total anthocyanin (**e**–**h** *labels* represent percent composition of total anthocyanin), phenotype (**i**–**l**). Conditions; control 0.5MS (**a, e, i**), pH 3.3 (**b, f, j**), AIC (**c, g, k**), 100 mM MgSO_4_ (**d, h, l**). *Scale* 600 µm

To answer this question, we conducted HPLC–PAD on aqueous methanol extracts from seedlings grown under the seven stress conditions. Again, AIC was used as a reference. Interestingly, different stresses were found to lead to distinct anthocyanin profiles (Fig. 3). Under control growth conditions, only four anthocyanins were detected: A8, A9*, A11* and A11 (Fig. 3a). A11, the most decorated anthocyanin, was the predominant anthocyanin, similar to that reported previously for wild-type *Arabidopsis* seedlings grown on agar- or vermiculite-based media (Tohge et al. 2005; Rowan et al. 2009). For seedlings grown on 100 mM MgSO_4_, we observed the *de novo* accumulation of A5*/A9, A5, A7, and A3, in addition to a moderate (less than tenfold) increase of the four anthocyanins found in the control (Fig. 3d). The *de novo* induction of several anthocyanin compounds in the MgSO_4_ condition was accompanied by a *circa* 10 % decrease in the relative proportions of A11 and A11* in the total anthocyanin content, compared to the control (compare Fig. 3h to Fig. 3e). In contrast, the proportions of A11 and A11* were 10 % greater and 10 % less, respectively, in the pH 3.3 condition compared to the control (compare Fig. 3f to Fig. 3e). The pH 3.3 condition also led to the *de novo* induction of A5*/A9, A5, A7, and A3, in addition to A8*, an anthocyanin not found in the MgSO_4_ condition. AIC had roughly 10- and 1.5-fold less induction of A11 compared to pH 3.3 and MgSO_4_, respectively. Seedlings grown under AIC had more than 80-fold increase of A9* compared to the control (Fig. 3c). A calculation of extinction coefficients (absorptivities) of purified A11 (61,300 L cm^−1^ mol) and A9* (53,400 L cm^−1^ mol) indicated that the induction by AIC rendered A9* levels equivalent to A11. AIC also resulted in a significant induction of A5 (*P* < 0.05, two sided *t* test), which was absent from the control and is generally not highly induced by the other stress conditions, and was the only condition that accumulated detectable levels of A3 (Supplemental Fig. S2). Overall, these results show that different stress conditions lead to unique anthocyanin profiles.

### Similar anthocyanin fingerprints for similar physiological stresses

**Fig. 4:**
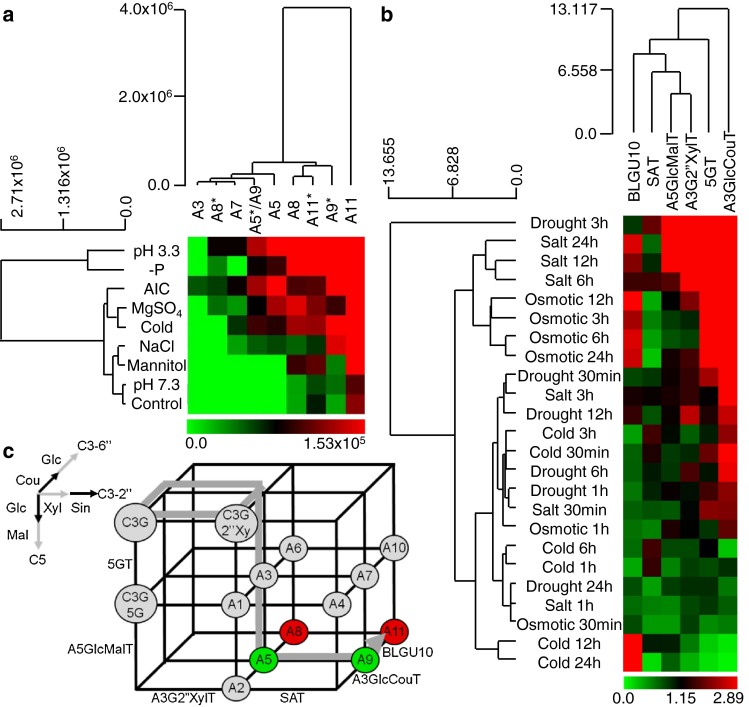
Clustering of stress responses by anthocyanin metabolite or gene profiles. Hierarchical clustering of stresses by anthocyanin metabolite profiles (**a**), or by gene expression profiles (**b**). A schematic representation of the anthocyanin biosynthesis grid in *Arabidopsis* (**c**), adapted from (Yonekura-Sakakibara et al. 2012). A5 and A9* metabolites are labeled *green*, and A8 and A11 *red*, to emphasize similar induction profiles in Fig. 5. *5GT* (At4g14090); *A5GlcMalT* (At3g29590); *A3G2″XylT* (At5g54060); *A3GlcCouT* (At1g03495), *SAT* (At2g23000); *BLGU10* (At4g27830)

As different subsets of anthocyanins showed different accumulation profiles in response to particular stresses, our next objective was to determine whether anthocyanin profiles could be used to identify similarities among stress responses, with the ultimate goal of establishing whether anthocyanin profiles can provide a “fingerprint” of the stress status of plants. To test this hypothesis, we performed hierarchical clustering using anthocyanin profiles (Fig. 4a). As expected, the anthocyanin profiles of control samples clustered with those grown in pH 7.3 medium. These two conditions led to the accumulation of the same four anthocyanins but with different relative amounts of the individual species. Osmotic stress (mannitol) clustered with NaCl. This is consistent with the fact that osmotic stress is a significant component of the overall stress caused by excess sodium ions (Hasegawa et al. 2000). Interestingly, MgSO_4_ clustered more closely with cold and AIC, than to NaCl. Roughly, ten percent of the genes up-regulated by cold were also found to be up-regulated by high salinity in a microarray containing about 7,000 independent full-length cDNAs (Seki et al. 2002). However, cold has been shown to induce anthocyanin synthesis and MgSO_4_ has been shown to stabilize anthocyanins (Shaked-Sachray et al. 2002), so the similarities in anthocyanin profiles in this case may be due to different mechanisms. Anthocyanin profiles from low pH (pH 3.3) and phosphate deficient conditions cluster together. This is consistent with the fact that phosphate in the medium becomes insoluble at low pH, and thus cannot be taken up by the plant (Hoeft et al. 2000). Notably, the −P and low pH treatments form a subcluster that is distinct from the other osmotic and high salinity stresses. Taken together, these results demonstrate that similar anthocyanin fingerprints are induced by related physiological stress conditions.

### Stress-induced versus constitutive anthocyanins

The hierarchical clustering of the different anthocyanins across stresses showed that A11 is a unique outlier (Fig. 4a). A11 accumulated to relatively high levels even in the absence of abiotic stress. The cluster containing A8, A9*, and A11* accumulated in stress and non-stressed conditions, and generally was induced most highly by stress. Members of the final cluster, comprised of A3, A5, A5*/A9, A7, and A8*, were exclusively induced by stress. These results show that there exists both stress inducible and constitutive (or developmentally induced) anthocyanin populations in *Arabidopsis*.

### Subsets of anthocyanins are similarly induced by a range of stress conditions

**Fig. 5:**
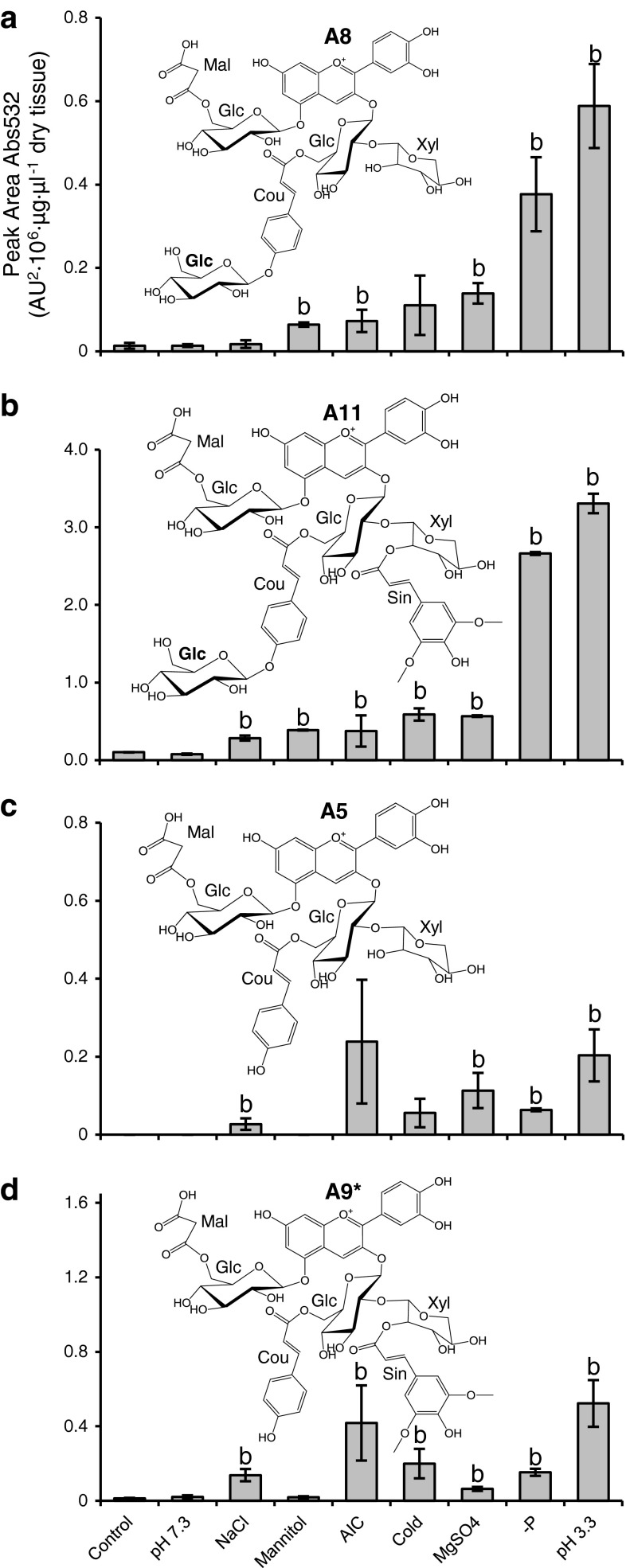
Levels of selected anthocyanins in different stress conditions. A8 (**a**), A11 (**b**), A5 (**c**), A9* (**d**). *Error bars* represent standard error of the mean (*n* = 3). ^a^Less than control, ^b^greater than control, *P* < 0.10; two-tailed Student’s *t* test

In light of the fact that stress conditions preferentially induce specific anthocyanins, we wanted to determine whether specific anthocyanin compounds show similar induction profiles across stress conditions, as this may suggest similar functional demand for particular sets of anthocyanins during stress, and/or co-induction of specific steps in anthocyanin biosynthesis. An analysis of the relative levels of single anthocyanins across the different stresses demonstrated that A8 had similar relative accumulation profiles as A11, with maximum levels found in seedlings deprived of phosphate and seedlings exposed to low pH (Fig. 5a, b). By contrast, A5 and A9* exhibited similar induction profiles, distinct from those of A8 and A11, with maximum levels found in AIC and −P (Fig. 5c, d). These two sets of anthocyanins differ in structure by the presence or absence of the glucose moiety attached to the coumaryl at position C3-6″ (position R2 in Fig. 1). The enzyme that catalyzes the addition of this glucose was recently identified to be the acyl-glucose-dependent glucosyltransferase, BGLU10 (Miyahara et al. 2013).

Anthocyanin biosynthesis is believed to be controlled primarily at the level of transcription of the genes encoding biosynthetic enzymes (Koes et al. 2005; Tohge et al. 2005; Quattrocchio et al. 2006; Petroni and Tonelli 2011a). To determine whether the coordinated induction of anthocyanins by stress may be explained by co-induction of gene transcripts, we performed hierarchical cluster analysis of anthocyanin gene expressions across salt, drought, and cold stress conditions, using datasets available from the Bio-Analytic Resource (BAR) for Plant Biology (http://bar.utoronto.ca). The enzymes for anthocyanin modification are depicted in Fig. 4c. The cluster analysis shows that *5GT* and *A3GlcCouT* were outliers, and were the most constitutively expressed genes under these stresses (Fig. 4b), suggesting these enzymes may not be responsible for the dissimilar induction profiles of A5 and A9* versus A8 and A11 (Fig. 5), however, it is worth noting that the stress conditions used for gene expression analysis and those for metabolite analysis were not identical. *A3G2″XylT* and *A5GlcMalT* formed a subcluster, as their induction was less frequent than the other anthocyanin modification enzymes, but most commonly coordinated. *SAT* was less frequently co-induced with *A3G2″XylT* and *A5GlcMalT*, and *BGLU10* was even less frequently co-induced with *SAT*. The infrequent coordinated induction of *BGLU10* with other anthocyanin genes during stress is consistent with A5 and A9* (labeled green in Fig. 4c) having induction profiles similar to each other (Fig. 5), but different than A8 and A11 (Fig. 5, metabolites labeled red in Fig. 4c), as the transfer of glucose to position C3-6″ is the biosynthetic step that separates these two anthocyanin groups (Fig. 4c).

## Conclusions

Anthocyanins are specialized metabolites that often accumulate in vegetative tissues when plants are subjected to different types of stress conditions (Hatier and Gould 2008). Whether (and how) anthocyanins function as stress protective molecules have been a subject of debate (Gould 2004b); the most parsimonious explanation being that they play a role in protecting against the ROS that often accumulate during stress. Our studies show that *Arabidopsis* often responds to different types of abiotic stress conditions not only by increasing total levels of anthocyanins, but also by altering the profiles of anthocyanin accumulation (Figs. 3 and 4). Indeed, while some of the profile changes are likely a consequence of alterations in the degradation of some anthocyanins (e.g., MgSO_4_), changes in the expression of genes encoding many of the enzymes involved in the decoration of anthocyanins suggest that most of the effects are transcriptional. These results support that plants can preferentially synthesize different anthocyanins in response to distinct stresses, suggesting that the various decoration patterns on an anthocyanin backbone actually impart a function favorable in a particular stress condition.

## Electronic supplementary material

Below is the link to the electronic supplementary material.

Supplemental Fig. S1 Amount of total flavonols and sinapate esters produced by *Arabidopsis* after various stress conditions. Plants were cultured under stress conditions, tissues were extracted, and metabolites analyzed as described in the Materials and Methods. Error bars represent standard error of the mean (*n* = 3). ^a^Less than control, ^b^greater than control, *P* < 0.05; two-tailed Student’s *t* test (PDF 18 kb)

Supplemental Fig. S2 Anthocyanin profiles of *Arabidopsis* seedlings under stress. Error bars represent standard error of the mean (*n* = 3). ^a^Less than control, ^b^greater than control, *P* < 0.05; two-tailed Student’s *t* test (PDF 54 kb)

## Abbreviations

5GT: Anthocyanin 5-*O*-glucosyltransferase
A5GlcMalT: Anthocyanin 5-*O*-glucoside-6″-*O*-malonyltransferase
A3G2″XylT: Anthocyanin 3-*O*-glucoside: 2″-O-xylosyltransferase
A3GlcCouT: Anthocyanin 3-*O*-glucoside: 6″-O-p-coumaroyltransferase
AIC: Anthocyanin inductive condition
BLGU10: Anthocyanin 3-*O*-6″-coumaroylglucoside: glycosyltransferase
HPLC–PDA: High performance liquid chromatography–photodiode array
LC–MS/MS: Liquid chromatography–tandem mass spectrometry
MS: Murashige and Skoog
−P: Without phosphate
PAP1: Production of anthocyanin pigment 1
ROS: Reactive oxygen species
SAT: Sinapoyl-Glc:anthocyanin acyltransferase
SE: Sinapate ester
