# Supplementary figures and images for: Not all anthocyanins are born equal: distinct patterns induced by stress in Arabidopsis
Source: Planta. 2014 Jun 6;240(5):931–40. doi: 10.1007/s00425-014-2079-1 (PMC4200348; doi:10.1007/s00425-014-2079-1)

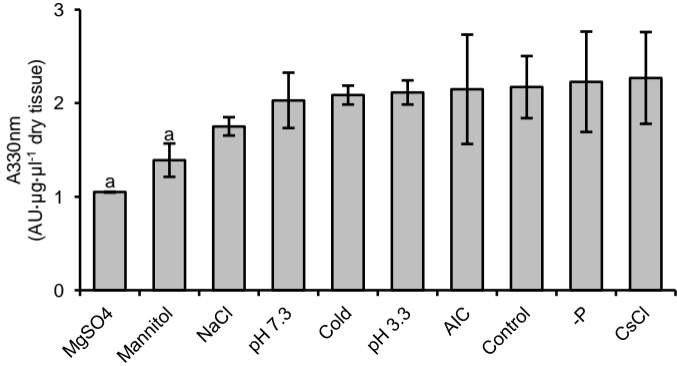

Supplement: Supplementary file 1 — Supplemental Fig. S1 Amount of total flavonols and sinapate esters produced by Arabidopsis after various stress conditions. Plants were cultured under stress conditions, tissues were extracted, and metabolites analyzed as described in the Materials and Methods. Error bars represent standard error of the mean (n = 3). aLess than control, bgreater than control, P < 0.05; two-tailed Student’s t test (PDF 18 kb) [file 425_2014_2079_MOESM1_ESM.pdf]

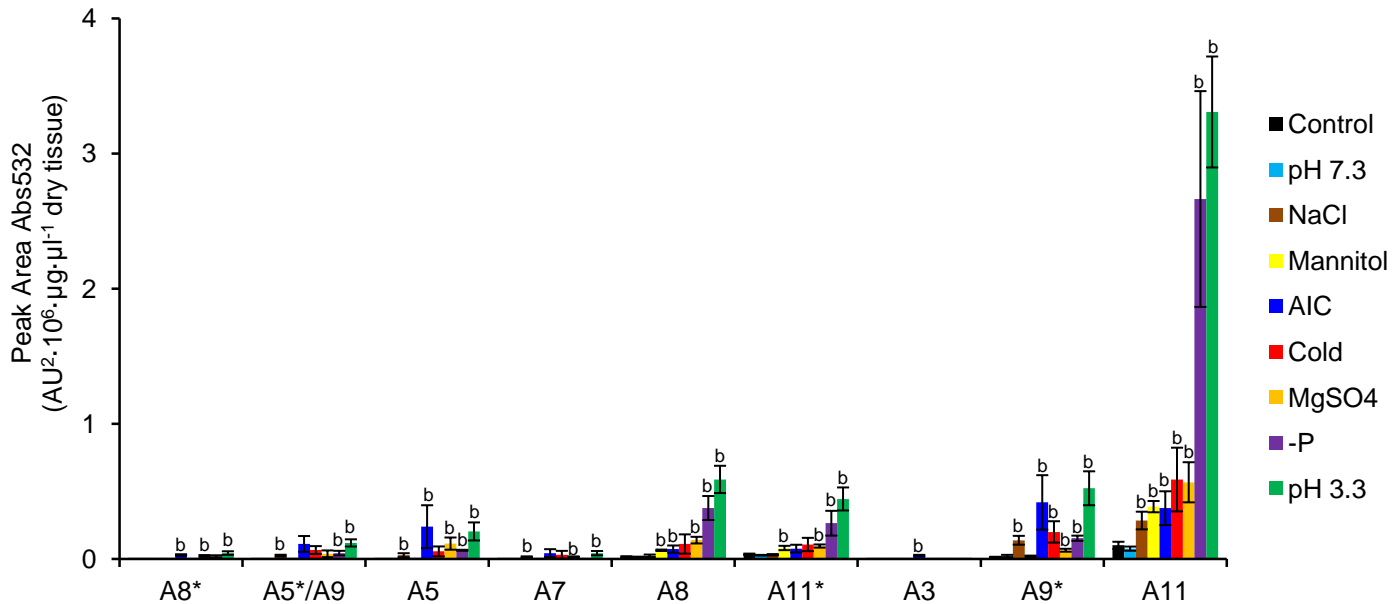

Supplement: Supplementary file 2 — Supplemental Fig. S2 Anthocyanin profiles of Arabidopsis seedlings under stress. Error bars represent standard error of the mean (n = 3). aLess than control, bgreater than control, P < 0.05; two-tailed Student’s t test (PDF 54 kb) [file 425_2014_2079_MOESM2_ESM.pdf]
